# Disparities in COVID-19 Vaccine Uptake Among Pregnant People in a Diverse Urban Population With High Vaccine Acceptance

**DOI:** 10.1016/j.focus.2024.100303

**Published:** 2024-11-22

**Authors:** Christine A. Blauvelt, Maura Jones Pullins, Stephanie L. Gaw

**Affiliations:** 1 Division of Maternal-Fetal Medicine, Department of Obstetrics, Gynecology, and Reproductive Sciences, University of California, San Francisco, San Francisco, California; 2 Division of Maternal-Fetal Medicine, Department of Obstetrics and Gynecology, University of North Carolina at Chapel Hill, Chapel Hill, North Carolina

**Keywords:** SARS-CoV-2, COVID-19, vaccines, immunization, vaccination hesitancy

## Abstract

**Introduction:**

This study's objective was to compare COVID-19 vaccination rates between pregnant individuals in San Francisco and the city's similarly aged population and to identify the factors associated with vaccine uptake in the pregnant population.

**Methods:**

This was a retrospective cohort study of all patients delivering at 2 hospitals in San Francisco from March 15, 2021, to October 15, 2021. Reference COVID-19 vaccination rates for San Francisco were obtained from the California Immunization Registry. The primary outcome was completion of a COVID-19 vaccine series among pregnant individuals compared with that in the San Francisco general population aged 18–45 years. Secondary outcomes included factors associated with COVID-19 vaccination among pregnant people.

**Results:**

Of 2,294 pregnant individuals, 1,181 (51.5%) completed a COVID-19 vaccine series by delivery. Pregnant individuals were less likely to be vaccinated than the San Francisco population throughout the study period. Factors associated with lower vaccination rates in pregnant individuals included public insurance (AOR=0.21, 95% CI=0.16, 0.27), younger age (AOR=0.74, 95% CI=0.64, 0.86), English language preference (AOR=0.58, 95% CI=0.42, 0.80), and self-identified Black (AOR=0.26, 95% CI=0.17, 0.40) (ref: White), Hispanic (AOR=0.61, 95% CI=0.46, 0.81), or other (AOR=0.50, 95% CI=0.36, 0.68) race or ethnicity.

**Conclusions:**

COVID-19 vaccination coverage among pregnant people in San Francisco lagged behind that of the city's similarly aged population, particularly among those with public insurance and of non-White race. Despite locally high vaccine acceptance, additional efforts are needed to address barriers and reduce disparities in COVID-19 vaccination within the pregnant population.

## HIGHLIGHTS

• COVID-19 vaccine uptake was lower in pregnant people than in the similarly aged city population.
• Individuals with public insurance and of non-White race had the lowest vaccination coverage.
• Addressing barriers to vaccination in low-income and Black, Indigenous, People of Color communities is essential.

## Graphical abstract

**Figure:**
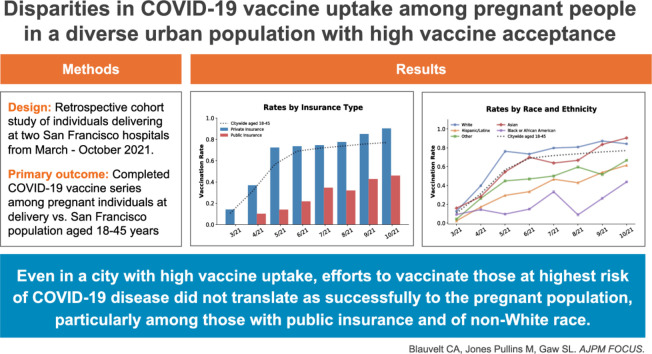

## INTRODUCTION

Vaccines preventing coronavirus disease 2019 (COVID-19) were a crucial tool for managing the global pandemic.^1^, ^2^, ^3^ Although pregnant patients were excluded from the preauthorization COVID-19 vaccine trials, they were eligible for vaccination once the vaccines were granted Emergency Use Authorization by the U.S. Food and Drug Administration on December 11, 2020.^4^ Pregnant patients were also prioritized in Phase 1c of the Centers for Disease Control and Prevention (CDC) vaccine distribution plan, which went into effect in California on March 15, 2021.^5^

Vaccine coverage among pregnant individuals is critically important given that COVID-19 during pregnancy is associated with worse clinical and pregnancy outcomes than in age-matched nonpregnant controls, including intensive care unit admission, mechanical ventilation, maternal death, stillbirth, and preterm birth.^6^, ^7^, ^8^ Major professional organizations, including the Society for Maternal-Fetal Medicine, the American College of Obstetricians and Gynecologists, and the CDC, recommend vaccinating pregnant individuals against COVID-19.^9^, ^10^, ^11^

Early in the pandemic, San Francisco was among the most highly vaccinated cities in the U.S., with 82% of eligible residents having received at least 1 dose of a COVID-19 vaccine by October 2021, including 71% of Black residents and 92% of Hispanic or Latinx residents.^12^ The city enacted a robust vaccine outreach plan, including placement of large-volume vaccination sites in the most affected communities, mobile vaccination clinics, free transportation to and from vaccination sites, and strategic partnerships with community-based organizations.^13^ Despite these successes, it is not known whether pregnant individuals in San Francisco were receiving the COVID-19 vaccine at similar rates as the general city population. The authors report COVID-19 vaccination rates among pregnant individuals delivering at 2 hospitals in San Francisco compared with vaccination rates of the San Francisco similarly aged population.

## METHODS

### Study Population

The authors performed a retrospective cohort study of all individuals who delivered at 2 academic hospitals in San Francisco (University of California San Francisco Benioff Children's Hospital and Zuckerberg San Francisco General Hospital) between March 15, 2021, and October 15, 2021. Study subjects were identified through the institutions’ electronic medical and delivery records. Detailed medical records review was performed to abstract demographic characteristics, clinical characteristics, and immunization status. Demographic characteristics included maternal age at delivery, self-reported race and ethnicity, primary insurance payer, and language preference. Race and ethnicity were included in this study because minoritized racial and ethnic groups may experience disparities in COVID-19 vaccination. Clinical characteristics included gestational age at delivery; parity; and medical conditions that increase the risk of severe COVID-19 disease, including diabetes, obesity (BMI ≥30 kg/m^2^), heart disease, lung disease, or immunocompromised state such as living with HIV or being on immunosuppressive medications. Immunization records included receipt of a COVID-19 vaccine, dates of vaccine administration, and vaccine type (BNT162b2 [Pfizer–BioNTech], mRNA-1273 [Moderna], or Janssen Ad26.COV2.S [Johnson & Johnson]).

The authors used the San Francisco Department of Public Health open-source platform DataSF linked to the California Department of Public Health California Immunization Registry (CAIR2) database to compare vaccination rates between the study's pregnant population and the San Francisco population aged 18–45 years.^12^ This database contains COVID-19 vaccination rates among San Francisco residents, including data stratified by age, race, and ethnicity. Data are not available on sex. All providers enrolled in the California COVID-19 Vaccination Program are required to report to CAIR2, thus COVID-19 vaccination data in CAIR2 are thought to be robust.^14^ The CAIR2 database was used to obtain vaccination data for both the pregnant population and the general city population.

### Measures

The primary outcome was completion of a primary COVID-19 vaccine series prior to the delivery date. For individuals receiving a BNT162b2 (Pfizer–BioNTech) or mRNA-1273 (Moderna) vaccine, this was defined as receipt of 2 vaccine doses. For individuals receiving a Janssen Ad26.COV2.S (Johnson & Johnson) vaccine, this was defined as receipt of 1 vaccine dose. Receipt of booster COVID-19 vaccines was not assessed in this study. Pregnant individuals were considered to have completed a primary COVID-19 vaccine series even if vaccines were administered prior to pregnancy. Secondary outcomes included factors associated with COVID-19 vaccination among pregnant individuals.

Vaccination rates for the population comparison cohort were calculated by dividing the number of San Francisco residents aged 18–45 years who completed a primary COVID-19 vaccine series by the population size estimate (*n*=405,156 for this age bracket).^12^ The population size estimate was obtained from the U.S. Census Bureau's American Community Survey for 2016–2020.^15^ Vaccination rates for the pregnant cohort were calculated by dividing the number of individuals who completed a COVID-19 vaccine series at delivery by the number of pregnant individuals delivering during the study period. Monthly vaccination rates were calculated by dividing the number of fully vaccinated pregnant individuals delivering in a given month by the total number of deliveries during the same period.

### Statistical Analysis

Descriptive analyses were performed for all demographic and clinical characteristics. Between-group differences were assessed by chi-square test or Fisher's exact test for binary variables and by *t*-test for continuous variables. Multivariable logistic regression analysis was performed to adjust for potential confounders. All demographic and clinical characteristics were included in the multivariable logistic regression analysis. Statistical analyses were performed using Python, Version 3.8.5 (Python Software Foundation, python.org); SciPy, Version 1.5.2 (SciPy.org); and pandas, Version 1.1.2 (pandas.pydata.org). All statistical tests were 2 tailed, with *p*<0.05 statistically significant. The University of California, San Francisco IRB approved this study. The STROBE reporting guideline was followed in the writing of this report.^15^

## RESULTS

A total of 2,294 pregnant individuals were included in this study. The mean (SD) age at delivery was 33.4 (6.4) years, 51.3% were nulliparous, and most self-identified as non-Hispanic White (33.6%) or Hispanic/Latinx (28.2%). Full COVID-19 vaccination by the time of delivery occurred for 1,181 individuals (51.5%). Receipt of at least 1 dose of a COVID-19 vaccine occurred for 1,338 individuals (58.3%) by delivery and 1,639 individuals (71.4%) by 6 weeks postpartum.

Table 1 summarizes the baseline characteristics of pregnant individuals in this study. Those fully vaccinated against COVID-19 at delivery were more likely to be older (mean [SD] age=35.0 [5.1] years vs 31.7 [7.1] years, *p*<0.001), be nulliparous (54.4% vs 47.0%, *p*<0.001), be primarily English speaking (89.0% vs 78.0%, *p*<0.001), self-identify as non-Hispanic White (44.3% vs 22.3%, *p*<0.001) or non-Hispanic Asian (23.2% vs 15.8%, *p*<0.001), and be immunocompromised (6.1% vs 2.8%, *p*<0.001) than individuals who were not fully vaccinated against COVID-19 at delivery. Vaccinated individuals were less likely to be publicly insured (18.5% vs 56.3%, *p*<0.001) or to have obesity (5.1% vs 9.4%, *p*<0.001) than unvaccinated individuals.

**Table 1:** Baseline Characteristics of the Pregnant Population According to Vaccination Status

Characteristic	Overall (N=2,294)	Vaccinated (*n*=1181)	Unvaccinated (*n*=1,113)	*p-*value
Age at delivery, years, mean ± SD	33.4 ± 6.4	35.0 ± 5.1	31.7 ± 7.1	<0.001
Gestational age at delivery, weeks, mean ± SD	38.0 ± 3.3	38.3 ± 3.1	37.8 ± 3.5	0.001
Nulliparous	1,177 (51.3)	654 (54.4)	523 (47.0)	<0.001
Race and ethnicity				
Asian	450 (19.6)	274 (23.2)	176 (15.8)	<0.001
Black	175 (7.6)	35 (3.0)	140 (12.6)	<0.001
Hispanic/Latinx	646 (28.2)	239 (20.2)	407 (36.6)	<0.001
Other	252 (11.0)	110 (9.3)	142 (12.8)	0.009
White	771 (33.6)	523 (44.3)	248 (22.3)	<0.001
Primary insurance payer				
Public insurance	845 (36.8)	218 (18.5)	627 (56.3)	<0.001
Private insurance	1,445 (63.0)	963 (81.5)	482 (43.3)	<0.001
None or unknown	4 (0.2)	0 (0.0)	4 (0.4)	0.12
Non-English primary language	375 (16.3)	130 (11.0)	245 (22.0)	<0.001
High-risk medical condition	625 (27.2)	303 (25.7)	322 (28.9)	0.09
Diabetes	342 (14.9)	162 (13.7)	180 (16.1)	0.11
Obesity	165 (7.2)	60 (5.1)	105 (9.4)	<0.001
Heart disease	54 (2.4)	28 (2.4)	26 (2.3)	1.00
Lung disease	91 (4.0)	44 (3.7)	47 (4.2)	0.59
Immunocompromised state	103 (4.5)	72 (6.1)	31 (2.8)	<0.001
Gestational age at first COVID-19 vaccine dose, weeks, mean ± SD^a^	—	21.1 ± 9.3	—	—
Vaccine type				
Pfizer–BioNTech	—	1238 (75.1)	—	—
Moderna	—	343 (20.8)	—	—
Johnson & Johnson	—	67 (4.1)	—	—

*Note:* All units are *n* (%) unless otherwise specified.

a Gestational age at first dose of COVID-19 vaccine was only calculated for individuals who received the first dose during pregnancy (*n*=1,153).

**Figure 1:**
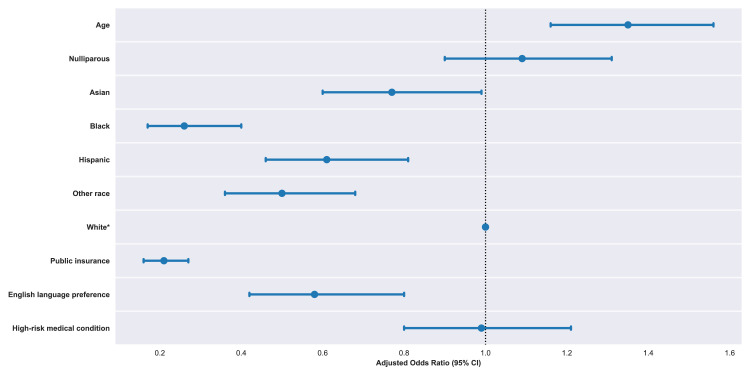
Characteristics associated with COVID-19 vaccination among pregnant individuals. *Note*: Data presented are AORs (95% CIs) calculated from a multivariable logistic regression. *White is the ref for the covariates Asian, Black, Hispanic, other, and White race and ethnicity.

In a multivariable logistic regression analysis, factors associated with lower COVID-19 vaccination rates at delivery included public insurance (AOR=0.21, 95% CI=0.16, 0.27), younger age (AOR=0.74, 95% CI=0.64, 0.86), English primary language (AOR=0.58, 95% CI=0.42, 0.80), and self-identified Black (AOR=0.26, 95% CI=0.17, 0.40; ref: White), Hispanic (AOR=0.61, 95% CI=0.46, 0.81) or other (AOR=0.50, 95% CI=0.36, 0.68) race or ethnicity (Figure 1). The presence of a medical condition associated with higher risk for severe COVID-19 (AOR=0.99, 95% CI=0.80, 1.21) and nulliparity (AOR=1.09, 95% CI=0.90, 1.31) were not associated with vaccine uptake.

**Figure 2:**
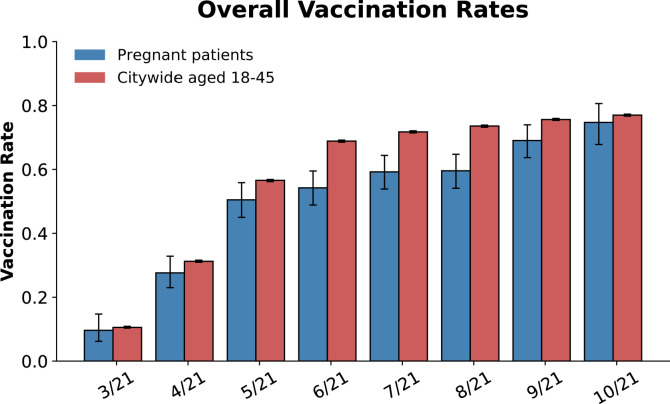
COVID-19 vaccination rates over time according to pregnancy status. *Note*: Overall COVID-19 vaccination rates among pregnant individuals (blue) versus the general San Francisco population aged 18–45 years (red) are presented. Error bars represent the 95% CIs for proportions calculated using the Wilson score interval method.

**Figure 3:**
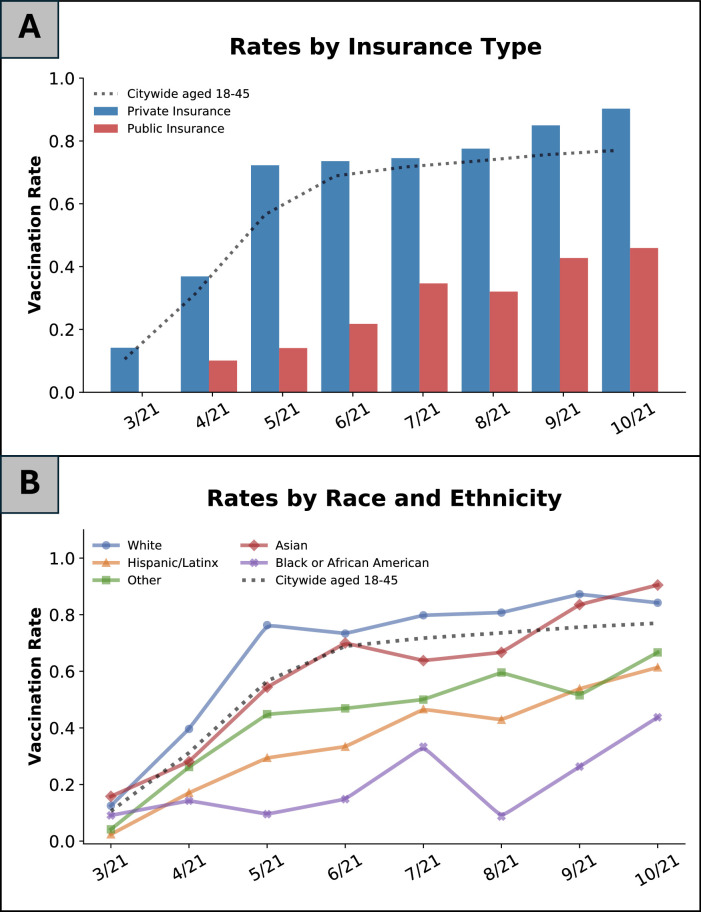
COVID-19 vaccination rates over time according to primary insurance payer and race and ethnicity. (A) COVID-19 vaccination rates among pregnant individuals with private insurance (blue) versus public insurance (red). (B) COVID-19 vaccination rates among pregnant individuals by race and ethnicity. *Note*: Dashed lines represent vaccination rates among the San Francisco general population aged 18–45 years.

Although vaccination rates increased over time for both the pregnant population and the overall San Francisco population, pregnant patients were less likely to be vaccinated at the time of delivery than San Francisco residents aged 18–45 years throughout the study period, with statistically significant differences from May 2021 through September 2021 (Figure 2). The authors used chi-square tests to perform month-by-month comparisons of COVID-19 vaccinations rates between the pregnant population and the general population: March (9.6% vs 10.5%, *p*=0.681), April (27.6% vs 31.2%, *p*=0.167), May (50.5% vs 56.6%, *p*=0.027), June (54.2% vs 68.9%, *p*<0.001), July (59.2% vs 71.7%, *p*<0.001), August (59.6% vs 73.6%, *p*<0.001), September (69.0% vs 75.6%, *p*=0.007), and October (74.7% vs 77.0%, *p*=0.474). After applying the Bonferroni correction for multiple comparisons (8 comparisons, thus threshold for significance=0.00625), the authors observed that only June, July, and August 2021 remained significant. Pregnant individuals with public insurance were less likely to be vaccinated than those with private insurance (25.8% vs 66.5%, *p*<0.01) (Figure 3A). Those identifying as Black, Hispanic, or other race or ethnicity had lower vaccination rates than White and Asian pregnant people and nonpregnant San Francisco residents throughout the study period (Figure 3B).

## DISCUSSION

This study demonstrates that COVID-19 vaccination coverage among pregnant individuals in San Francisco lagged behind that of the city's general population aged 18–45 years. Some groups exhibited dramatically lower vaccination rates. Public insurance was associated with a nearly 5-fold decreased odds of COVID-19 vaccination, and this gap persisted throughout the study period. Non-White birthing people in this study were also significantly less likely to have received a COVID-19 vaccine, notably with those identifying as non-Hispanic Black having more than 3-fold decreased odds of vaccination. Other factors associated with lower likelihood to be vaccinated included younger age and English language preference. There was no increased vaccine uptake in those with comorbid medical conditions associated with severe COVID-19 disease.

When COVID-19 vaccines initially became available, there were limited data on vaccine safety and efficacy during pregnancy due to the exclusion of pregnant persons from preauthorization clinical trials. Over time, there has been a growing body of evidence demonstrating that COVID-19 vaccines are highly effective in pregnant patients without safety signals, including miscarriage or fetal anomalies.^16^, ^17^, ^18^, ^19^ Pregnant individuals with COVID-19 infection are also at higher risk for severe COVID-19 disease and adverse obstetric outcomes.^6^, ^7^, ^8^ However, the lag in the quality and size of COVID-19 vaccination data among pregnant populations, along with rampant spread of vaccine misinformation, likely contributed to decreased confidence in their safety and efficacy.

This study also confirms prior work that socioeconomic factors were associated with differential vaccine coverage among pregnant persons.^20^ Socially vulnerable communities have significant and compounding barriers to healthcare access, including challenges with transportation, childcare, and time off work for appointments or illness. Moreover, a long history of systemic racism and unethical research on Black, Indigenous, and People of Color and immigrants likely contributes to vaccine mistrust.^21^ Improving accessibility and acceptability of COVID-19 vaccines among low-income communities and communities of color is a crucial aspect of addressing inequities in COVID-19 outcomes.

The authors report higher overall vaccination rates in San Francisco pregnancies than published data by the CDC, which found only 34.4% vaccination coverage among pregnant individuals in the U.S. as of October 2021.^12^^,^^22^ Factors that may have contributed to this include high vaccine acceptance among the general San Francisco population and state and citywide initiatives to encourage COVID-19 vaccination.^13^ The San Francisco Department of Public Health established vaccine access sites across the city at community clinics, neighborhood sites, and mobile vaccination teams as early as January 2021. San Francisco also offered free transportation for those getting a COVID-19 vaccine. Finally, vaccine mandates were implemented for access to restaurants and gyms and for employment for healthcare workers, teachers, and state employees. Despite this, the pregnant cohort had lower vaccination rates than citywide rates, suggesting that efforts to vaccinate those at highest risk of COVID-19 disease did not translate as successfully to the pregnant population.

Outbreaks of COVID-19 fueled by variants will require ongoing use of vaccines and booster shots. Moreover, new vaccines are being developed for use in pregnancy such as the vaccine for respiratory syncytial virus to enhance infant immunity in the first few months of life.^23^ Research into vaccine uptake in pregnant populations is essential for guiding public health strategies. Moving forward, pregnant persons should be prioritized in high-quality research, including clinical trials evaluating the safety and efficacy of vaccines.^24^^,^^25^ Programs should also be developed to increase the capacity of healthcare professionals to engage in vaccine counseling and to address pregnant patients’ vaccine hesitancy. Prior studies have demonstrated that provider recommendation about COVID-19 vaccination is predictive of vaccine uptake.^26^^,^^27^ In addition, community partnerships and sustained efforts to understand and respond to the needs of historically marginalized communities are essential to addressing disparities in outcomes associated with both pregnancy and COVID-19 disease.

Strengths of this study included comparing COVID-19 vaccine rates between pregnant persons and the similarly aged local population, which revealed that the overall population rates may not be representative of vaccination in pregnancy. In addition, this study investigates COVID-19 vaccination rates among a population not well represented in the CDC Vaccine Safety Datalink.^22^ The study cohort was derived from patients from 2 academic hospitals (1 tertiary referral hospital and 1 public safety-net hospital) with varied insurance status, in contrast to the large vertically integrated healthcare systems comprising the Vaccine Safety Datalink.

### Limitations

Several limitations of this work should be recognized. The authors only included data on pregnant persons delivering at 2 university-affiliated labor and delivery units; thus, this study may not be representative of the larger pregnant population of San Francisco. In addition, the comparison population included both male and female residents of San Francisco, whereas the pregnant population contained only female individuals. The authors were also unable to exclude pregnant individuals from the comparison population; thus, most of the pregnant cohort was likely contained within the general population cohort. Although the CAIR2 vaccine database is thought to be reliable for COVID-19 vaccines administered in California, it does not capture vaccines administered outside of California. Some individuals may also have missing vaccination data due to a lag between input of vaccine records into CAIR2 and this study's data pull (11/15/2021). CAIR2 mandates that vaccine records be submitted within 14 days of administration; thus, the authors do not anticipate that a data lag affected a significant proportion of the study population. The authors only included variables that were accessible in the electronic medical record; however, some variables such as number of prenatal visits and provider recommendation to receive a COVID-19 vaccine were not available and thus not included in this study. Finally, this study does not assess drivers of COVID-19 vaccine hesitancy or provide actionable steps for reducing disparities in vaccination rates among vulnerable populations, including those with public insurance and communities of color. This remains an important topic for future work.

## CONCLUSIONS

COVID-19 vaccination coverage among pregnant people in San Francisco lagged behind that of the city's similarly aged population, particularly among those with public insurance and of non-White race. Despite locally high vaccine acceptance, additional efforts are needed to address barriers and reduce disparities in COVID-19 vaccination within the pregnant population.
